# Advanced phenotyping and phenotype data analysis for the study of plant growth and development

**DOI:** 10.3389/fpls.2015.00619

**Published:** 2015-08-10

**Authors:** Md. Matiur Rahaman, Dijun Chen, Zeeshan Gillani, Christian Klukas, Ming Chen

**Affiliations:** ^1^Department of Bioinformatics, College of Life Sciences, Zhejiang University, HangzhouChina; ^2^Leibniz Institute of Plant Genetics and Crop Plant Research, GaterslebenGermany

**Keywords:** plant phenotype, high-throughput phenotyping, environmental factor, imaging procedure, data analysis

## Abstract

Due to an increase in the consumption of food, feed, fuel and to meet global food security needs for the rapidly growing human population, there is a necessity to breed high yielding crops that can adapt to the future climate changes, particularly in developing countries. To solve these global challenges, novel approaches are required to identify quantitative phenotypes and to explain the genetic basis of agriculturally important traits. These advances will facilitate the screening of germplasm with high performance characteristics in resource-limited environments. Recently, plant phenomics has offered and integrated a suite of new technologies, and we are on a path to improve the description of complex plant phenotypes. High-throughput phenotyping platforms have also been developed that capture phenotype data from plants in a non-destructive manner. In this review, we discuss recent developments of high-throughput plant phenotyping infrastructure including imaging techniques and corresponding principles for phenotype data analysis.

## Introduction

Global agricultural demand is expanding rapidly, not the least because of a growing world population but also due to indirect factors which are rendering agricultural production suboptimal, such as unequal food distribution, competing claims for land use and increased demand for meat and dairy due to a change in dietary habits in the G5 countries (emerging economics). Agriculture, in particular, faces tremendous challenges for crop production in the coming decades. According to a prediction by the United Nations Food and Agriculture Organization^1^, cereal production must be doubled before 2050 to satisfy the demand for food by the growing world population, as well as the increasing competition for crops as sources of bio-energy, fiber, and other industrial purposes. Additionally, the supply of the major crop rice, a staple food throughout the world, has become insufficient (Furbank et al., 2009). Besides the many biotic and abiotic factors, predicted changes in temperature and rainfall patterns as a consequence of climate change may lead to further reduction in yields (Sticklen, 2007). In order to meet the global challenges represented by the rapidly growing human population and environmental changes, novel methods are required to improve the quality and productivity of cereal grains (Tester and Langridge, 2010). For these reasons, there is a demand for quantitative analyses of plant traits to accelerate the selection of crops that are better adapted to resource limited environments and soil conditions, which is also a major constraint to global food production (Fiorani and Schurr, 2013).

Plant researchers have been trying to propose appropriate strategies for plants that will be resistant to environmental stress, insects and diseases, while still possessing high nutrient efficiency (Zhang, 2007; Ahmed et al., 2013). As a way to improve cereal crops, a lot of effort has been put into functional genomics studies using high-throughput genomic tools (Ayliffe and Lagudah, 2004; Xing and Zhang, 2010; Huang et al., 2013; Pallotta et al., 2014; Valluru et al., 2014). However, more effort is required to map genotype-phenotype relationships for the global development of crop breeding (Tester and Langridge, 2010). Due to the rapid progression of functional genomics and genetic technologies, especially in the field of high-throughput sequencing technology many plant genomes are now available. Their functional analysis has entered the high-throughput phase, providing available genetic information as well as enabling genomic analysis (Holtorf et al., 2002). These sequenced genomes that are supposed to represent a crop species often only constitute a single genotype.

The outdated phenotyping procedures- a technique dealing with plant characteristics, in conjunction with available genetic information, have not allowed a thorough functional analysis and have not led to a functional map between genotype and phenotype. A focus on overcoming these shortcomings has led to an emerging and increasingly important branch of biological sciences termed “phenomics” (Furbank, 2009; Furbank and Tester, 2011). Phenomics is a technology that enables high-throughput phenotyping for crop improvement in response to present and future demographic and climate scenarios.

To meet the needs of current research, reliable, automatic and high-throughput phenotyping platforms have been developed (Hartmann et al., 2011). Multiple studies in phenomics highlight findings, such as causal genes and background variation, relationships between traits, plant growth behavior as well as reproduction in various conditions (Furbank and Tester, 2011; Yang et al., 2013; Brown et al., 2014). In this way, the challenges of extracting multi-parametric phenotypic information along with the genetic variability can be adequately met. Current phenotyping platforms include a variety of imaging methodologies to obtain high-throughput non-destructive phenotype data for quantitative studies of complex traits, such as growth, tolerance, resistance, architecture, physiology, yield, and the basic measurement of individual quantitative parameters that form the basis for more complex traits (Chen et al., 2014b; Li et al., 2014).

High-throughput automated imaging is now the ideal tool for phenotyping, and is becoming more advanced and popular, with the capacity to measure multiple morphological and physiological traits for an individual plant. There is also a trade-off in speed versus accuracy when using high-throughput imaging- manual measurement of several traits on one plant, if done properly, is currently more accurate than automated measurement, but much slower. Furthermore, through imaging techniques, plant phenomics could offer plant scientists a new way to discover the features and functionality of living plants via scanning temperature profiles, measuring photosynthetic rates, gauging growth rates, and getting insight into root physiology (Finkel, 2009). These advances have also boosted plant phenotyping to a new level. Therefore, we describe phenotyping techniques based on various imaging systems. In addition, we highlight the importance of phenotype data analysis, analytical techniques, and methods for plant growth and developmental studies. We also highlight the major challenges of high-throughput phenotyping and phenotype data analysis for promising applications in plant phenomics.

## Consequences of Environmental Factors for Plant Phenotyping: A Big Challenge for the Imminent Generation

Several studies have suggested that upcoming generations can be influenced by the environmental factors experienced by the earlier generation (Dawson et al., 2011). Recent studies indicate that under rapid climate change phenotypic plasticity rather than genetic diversity is more likely to play a crucial role in allowing plants to persist in their environments (Vitasse et al., 2010; Gratani, 2014). The plant reacts by exhibiting phenotypic plasticity when the genotype is grown under various environmental conditions, and this plasticity is particularly big under extreme conditions such as frost, drought, and salinity.

The factor frost is one of the most important abiotic stresses for the countries with severe winters, adversely affecting crop development and yield production (Chinnusamy et al., 2007; Li et al., 2011). Moreover, drought is a complex stress that permanently affects the soil, which elicits a wide variety of plant responses and limits crop yield (Pennisi, 2008; Honsdorf et al., 2014). This is a worldwide threat for agricultural production, and crop improvement of drought tolerance is a principal target. Soil salinity is another major abiotic stress that threatens sustainability of global crop production (Rengasamy, 2006). For instance, in Southern Asia and South East Asia about 48 million ha of potentially useful agricultural land is unusable due to saline soils (Hairmansis et al., 2014). Also, in those regions fertile land is often used to expand cities, and so crop production is decreasing significantly (Hedhly, 2011).

To assess the performance of plant species, it is crucial to increase the understanding about plant reactions to different stress environments and in which ways genotypes differ in such responses (Suter and Widmer, 2013). Moreover, for both economic and social importance, there is a need to know about the phenotype response to breed for increased yield and yield stability in the face of changing climate and environment (Brown et al., 2014). Hence, to improve crop production, it is necessary to link suitable phenotyping protocols in all stages, such as the screening of germplasm collections, mutant libraries, mapping populations, transgenic lines, breeding materials, and the design of “omics” and QTLs experiments (Salekdeh et al., 2009). Scientists are using advanced approaches to explain genetic mechanisms underlying the major plant phenotypic traits (Salekdeh et al., 2009). Furthermore, additional exploration and use of the novel plant development approaches are urgently required for the imminent challenging decade.

## Importance of Advanced Phenotyping and Phenomics in Modern Agriculture

With the rapid development of sequencing technologies, whole genomes of many plant species are now available in online databases. The sequencing of the genome of the model plant *Arabidopsis* represented a landmark in plant genomics (Weigel and Mott, 2009). There are also many economically important crop varieties that have since been sequenced and annotated (Cannon et al., 2009; Weigel and Mott, 2009). However, making sense of, and exploiting genetic information for genomic analysis still requires considerable effort.

The selection of high yielding and stress-tolerant plants is necessary to ensure that crop production keeps pace with population growth. By establishing the connection between genotype and phenotype, it is possible to improve agricultural production to satisfy the requirement of the growing human population. Therefore, phenotyping is as important as genotyping in establishing the relationship between genes and traits. Indeed, phenotyping is rapidly becoming the major operational bottleneck in limiting the power of genetic analysis and genomic prediction.

Phenotyping tools in common use are labor-intensive, time-consuming and costly, and require destruction of plants at fixed times or at particular phenological stages. The goal of current plant phenotyping is to raise the accuracy, precision, and throughput of phenotype inference at all levels of biological organization, while reducing costs and labor through mechanization, remote sensing, improving data integration, and experimental design. However, with technological advances in plant breeding, genetic progresses through “omics” approaches are being conducted to meet the ideal phenotype, which will enable plants to have superior and stable yields under changes in climate and environment. These large-scale “omics” approaches are routinely used in various research disciplines of plants to study cellular processes, their genetic control and interactions with the environmental changes in molecular plant biology (Deshmukh et al., 2014).

The available components of “omics” approaches contain genomics, proteomics, transcriptomics, epigenomics, and metabolomics (Chen et al., 2014a). Integrated “omics” approaches have more potential in aiding crop breeding, leading to a new approach- “phenomics”- involving high-throughput analysis of physical and biochemical traits of an organism. The concept of phenomics has altered the strategy in crop development research, and it is defined as the study of phenome- the full set of phenotypes of an organism. In genomics, a sequenced genome is fully characterized, whereas in phenomics, we cannot characterize the entire phenome due to its highly dynamic and high-dimensional properties. However, we can carry out high-throughput and high-dimensional phenotyping of a set of particular traits. In plant phenotyping, throughput refers to the number of individual units at particular organizational levels within plants, and dimensionality refers to the diversity of phenotypic traits measured at various spatial and temporal regulations and in different categories, such as plant structure, physiology, and performance. Dimensionality also includes the number of genotypes and the diversity of environmental conditions and treatments taken into account upon phenotyping (Dhondt et al., 2013).

Genotype-phenotype mapping, along with the significant rate of trait discovery, has enormously improved phenotypic prediction (Topp et al., 2013). Integrated data from phenotype and genome-wide approaches provide models of the biological processes over time and across various scales. Quantitative trait loci (QTL) mapping and genome-wide association studies (GWAS) have been a useful tool for genetic analysis, giving valuable information about genomes in various plant studies. They have been broadly adopted for gene mapping (Yin et al., 2004; Atwell et al., 2010; Huang et al., 2010; Wurschum et al., 2011; Ranc et al., 2012; Wang et al., 2012; Topp et al., 2013). Comprehensive phenome- wide data enable plant similarity or dissimilarity to be studied across the whole population. Consequently, phenomics studies increasingly characterize all possible phenotypes, establishing the structural, physiological, and performance related traits (biomass/ha, seed yield) under different environmental conditions for a given genotype.

## Mechanism of Imaging Technologies: Meeting Challenges and Needs in Plant Phenomics

Imaging and image processing techniques with light sources from visible to near infrared spectrum provide non-destructive plant phenotype image datasets. These approaches have accelerated the precision and speed of real-time, high-throughput, and high-dimensional phenotype data for modeling and prediction of plant growth and structural development (Tardieu and Tuberosa, 2010; Golzarian et al., 2011). The application of combined image based novel technologies in phenomics and dedicated high-throughput dynamic controlled environment facilities have resulted in increased performance, and provide a new prospect for improving plant phenotype.

Materially, plant phenotyping is not a new research for recording quantitative and qualitative plant traits. It has been the backbone of most studies in ecology, agronomy, and eco-physiology to explore plant functional diversity, compare the performance of species, or study plant responses to the environment (Granier and Vile, 2014). Phenotyping has been progressing from the manual, non-destructive or destructive, study of a few different genotypes, which can only be done for a few replications. Non-destructive phenotyping is performed for intact plants; while destructive phenotyping is an invasive measurement where the plants can no longer be used for further experiments. The developmental course (kinetics) of the same organ cannot be monitored destructively. Basically, destructive measurements are more complicated, time consuming, and demand high labor costs. However, when measurements are carried out manually, non-destructive phenotyping can be even more time consuming and labor intensive.

The advanced imaging-based phenotyping procedure is ideal for combining controlled irrigation and phenotype protocols (Berger et al., 2010). They enable studies to establish potential heritable traits and understanding the complex regulatory networks underlying adaptive phenotypic variation on a population with fully sequenced genome in high-throughput quantitative studies (Cooper et al., 2009; Munns et al., 2010; Furbank and Tester, 2011). Imaging-based high-throughput plant phenotyping platform has led to popular tools for plant biology, underpinning the field of plant phenomics (Paproki et al., 2012). Various imaging methodologies, such as visible light imaging, infrared imaging, fluorescence imaging, imaging spectroscopy, etc., are being used to collect multi-level phenotype data from macroscopic to molecular scale over a few seconds to weeks (Sozzani et al., 2014).

Since imaging methodologies are the key technologies in plant phenomics with increasing importance, the main goal is to measure quantitative phenotype through the interaction between plants and light, such as reflected photons, absorbed photons, or transmitted photons. The best phenotyping practice also requires standardized experimental protocols, including imaging sensor calibration and a precise definition of raw data processing routines.

### Available Imaging Devices for High-Throughput Phenotyping

#### Visible Light Imaging

In plant science, visible light imaging has been broadly adopted due to its low cost and simplicity. Using this imaging system, with a similar wavelength (ranging from 400 to 700 nm) perception as the human eye, two-dimensional (2D) images can be used to analyze numerous phenotypic characteristics and to record the changes in plant’s biomass (Tackenberg, 2007; Bylesjo et al., 2008; Duan et al., 2011; Golzarian et al., 2011). To spread the spatial and volumetric information of phenotype images, three-dimensional (3D) imaging approaches have been developed, which could provide more accurate estimations of the morphological features (Clark et al., 2011; Paproki et al., 2012).

Therefore, during the integration of 2D and 3D image analysis, visible light imaging techniques are popular components for the integrated plant phenotyping platform (Yang et al., 2013). It represents raw data of a phenotype image in spatial matrices based on the intensity values relating to photon fluxes (red∼600 nm, green∼550 nm, blue∼450 nm) of the visible light spectral band. Although, it is the most trivial method in plant phenotyping, the drawback is that visible images only provide physiological information, and the common problem is created by the overlapping adjacent leaves and soil background during segmentation process (Fiorani and Schurr, 2013; Li et al., 2014).

#### Infrared Imaging

Infrared imaging technologies are used for screening objects of internal molecular movements which emit infrared radiation (Kastberger and Stachl, 2003). Two popular infrared imaging devices- a near-infrared (NIR) and a far-infrared (Far-IR, also called IR thermal)- can be used to screen radiation images. Many studies have combined visible and NIR imaging to detect vegetative indices due to the fact that healthy plants reflect a large proportion of NIR light (800–1400 nm), whereas soil reflects little NIR light. Moreover, soil and unhealthy plants reflect considerably more red light as compared with healthy plants (Yang et al., 2013).

The major advantage of visible light and NIR imaging are that they can assess plant health status response to different stress conditions. Visible and NIR digital imaging techniques are more suitable for screening multi-traits and nitrogen status under stress condition (Rajendran et al., 2009). For drought resistance, IR thermal imaging can be used to visualize temperature differences. A thermal infrared imaging technique has been introduced in both, laboratories and fields, and can characterize mutant screens, drought tolerance, salinity tolerance, osmotic tolerance, tissue tolerance, and Na^+^ exclusion. It can be used to compare chlorophyll pigments, leaf color and canopy temperature (Merlot et al., 2002; Jones et al., 2009; Munns et al., 2010). Infrared imaging has improved drought resistance and/or salinity resistance research by quantifying the osmotic tolerance in response to drought or salinity stress (Munns et al., 2010).

The benefits of the infrared imaging technologies are that they provide spatial resolution and more precise measurement under changing environmental conditions, and in field trials a large number of plots can be imaged at the same time (Li et al., 2014). One limitation of thermal imaging in the field is that it needs to include correction of soil background, wind impact and effects of transient cloudiness (Jones et al., 2009; Munns et al., 2010; Fiorani and Schurr, 2013).

#### Fluorescence Imaging

Fluorescence imaging is used from laboratory to field. This imaging technique describes the information about the plant metabolic status that can be obtained by the artificial excitation of the plant photo systems and observation of the relevant responses (Li et al., 2014). It is based on charge-couple device (CCD) cameras with sensitive fluorescence signals, where the signals occur by illuminating samples with visible or ultraviolet light. There are two types of fluorescence (red to far red region and the blue to green region) generated by the ultraviolet illumination ranging from 340 to 360 nm, and is expressed as a principle of underlying multi color fluorescence imaging. This technique offers the simultaneous capture of fluorescence emission, and provides a quick way to probe photosystem II status *in vivo* (Schreiber, 1986; Daley et al., 1989; Maxwell and Johnson, 2000; Baker, 2008).

There have been several uses of fluorescence imaging proposed for early detection of stress responses to biotic and abiotic factors before a decline in growth can be measured (Baker, 2008; Jansen et al., 2009; Konishi et al., 2009; Munns et al., 2010; De Smet et al., 2012; Chen et al., 2014b). To screen large mutant collections and to characterize mutants with different photosynthetic pigment composition, portable fluorometers, and fluorescence cameras are widely used (Niyogi et al., 1998; Lu et al., 2011). Furthermore, fluorescence imaging technique provides powerful diagnostic tool to resolve the heterogeneity problem of leaf photosynthetic performance, and is used in many areas of plant physiology (Baker, 2008). Most of the fluorescence imaging applications are limited to the seedling level or the single leaves of model crop. However, it is necessary to develop more robust software and standard procedures for the fluorescence image phenotyping, processing, and data analysis.

#### Spectroscopy Imaging

The use of spectroscopy imaging is very promising for plant phenotyping. It measures the interaction of solar radiation with plants, and originated from remote sensing of vegetation research (Kokaly et al., 2009; Li et al., 2014). Spectral measurements of the electromagnetic spectra can be obtained through multispectral or hyperspectral cameras that are capable of scanning wavebands of interest at high regulation (Fiorani and Schurr, 2013). Multispectral and hyperspectral measurements of the absorption band in the infrared range are used to describe various water statuses that estimate the canopy water content. The best usable examples of spectral measurements is the derivation of a number of reflectance vegetation indices from simple differences between two wavelength reflectance values to normalized reflectance values. The reflected spectra carry the information about plant architecture and health condition, which can be used to evaluate growth characteristics.

Beyond visible and infrared imaging methods, hyperspectral imaging method can divide images into bands, thus providing a huge portion of the electromagnetic spectrum of the images (Yang et al., 2013). The high spectral resolution of hyperspectral technologies make it an essential method for detecting the severity of damage caused by insects (Huang et al., 2012; Yang et al., 2013). The application of spectroscopy imaging is well-suited for field phenotyping when combined with aerial platforms, but the cost of the spectral cameras and its related infrastrucres are relatively expensive.

#### Structural Tomography and Other Imaging

In recent times, modern optical 3D structural tomography and functional imaging techniques have been developed and extended to improve living plant visualization. Functional imaging such as chlorophyll fluorescence imaging and PET (Positron emission tomograpy) are used for finding photosynthetic performance, stress, and focuses on physiological changes (Baker, 2008). The combination of structural tomography and functional imaging can screen more precise physiological activity of plant. Another novel imaging technique, MRI (magnetic resonance imaging) is used for imaging of internal physiological processes occurring *in vivo* (Borisjuk et al., 2012). Screening the dynamic changes in plant functions and structures by the combining technique of MRI and PET provides a novel functional and structural imaging procedure (Jahnke et al., 2009).

The FRET (Förster resonance energy transfer) sensor is another of the non-invasive advanced imaging technologies for high-resolution measurement of small molecules in living tissue based on genetically encoded, ratiometric fluorescent sensors that bind to and report on levels of the target molecule (Jones et al., 2014). It is used for molecular phenotyping, and a single FRET sensor can lead to discoveries of multiple pathways and processes involved in the dynamics of the sensor target. The cellular/subcellular location of interest has to be properly characterized and expressed by a FRET sensor, and measurements can be easily acquired with high temporal and spatial resolution (Okumoto et al., 2012). As the application example, FRET has been used in plant tissue to study calcium and zinc dynamics with subcellular spatial and real-time temporal resolution, the characterization of sugar transport in roots of insect seedlings, the identification of novel sugar transporters (Jones et al., 2014). To address many basic questions of plant growth and development, FRET could be an outstanding technology for advanced phenotyping.

Each of these digital photonics-based systems acquire phenotype image data from plant laboratories, greenhouse or fields, and monitors these with special imaging sensor via a remote system. **Table 1** illustrates a summary of optical photonics-based key techniques and applications in advanced phenotyping.

**Table 1:** Key of imaging techniques and applications purpose.

Imaging system	Description	Phenotypic trait parameters	Application purpose
Visible light	The visible light imaging technique is camera sensitive and produces gray or color scale images.	Image-based projected biomass, dynamic growth, color, shape descriptors, root architecture, seed morphology, panicle traits, etc.	This imaging technique can be used to assess plant growth status, biomass accumulation, nutritional status, or health status (Golzarian et al., 2011; Camargo et al., 2014; Yang et al., 2014).
Thermal infrared	Thermal infrared imaging sensor includes near-infrared, multispectral line scanning cameras. This imaging technique produces time series or single-time-point analysis based data.	Leaf area index, shoot or leaf temperature, surface temperature, insect infestation of grain, leaf and canopy water status, composition parameters for seeds, disease severity, etc.	This imaging technique used to characterize the plant temperature responses to the water status and transpiration rate and detect difference in stomatal conductance of the plant for adoption abiotic stress (Chen et al., 2014b).
Fluorescence	Fluorescence imaging technique detects chlorophyll and other fluorophores signals using fluorescence cameras.	Photosynthetic performance, quantum yield, non-photochemical quenching, leaf disease severity assessments, leaf health status, etc.	It provides a fleet way to probe photosystem status *in vivo*, diagnosing early stress responses before decline growth (Fiorani and Schurr, 2013), useful for disease detection in genetic disease resistance (Chen et al., 2014b), mapping QTLs for growth-related traits (El-Lithy et al., 2004), characterizing mutants with numerous photosynthetic pigment compositions (Niyogi et al., 1998), etc.
Hyperspectral	This imaging technique use hyper spectral, thermal cameras produced continuous, or discrete spectra raw data.	Water content, leaf growth and health status, panicle health status, grain quality, pigment composition, etc.	This imaging technique used to measure spatiotemporal growth patterns during the experiment and provide insight into the diversity of growth dynamics (Chen et al., 2014b).
CT	It is based on X-ray digital radiography/computed tomography.	Grain quality, tiller, morphometric parameters, water content, flow velocity, etc.	This imaging is widely used to asses tissue density (Aerts et al., 2014), measuring tiller numbers (Yang et al., 2014), grain quality, etc.
PET	Positron emission tomography.	Water transport, flow velocity, etc.	This is used to visualize distribution and transportation of radionuclide-labeled tracers involved in metabolism-related activities (Jahnke et al., 2009; Granier and Vile, 2014).
MRI	Magnetic resonance imaging.	Water content, morphometric parameters, etc.	The purpose of this imaging technique is to visualize metabolites, provides structural information, and monitor internal physiological processes occurring *in vivo* (Borisjuk et al., 2012; Granier and Vile, 2014).

### Experiment Setup and Large-Scale Phenotype Data Collection

**FIGURE 1:**
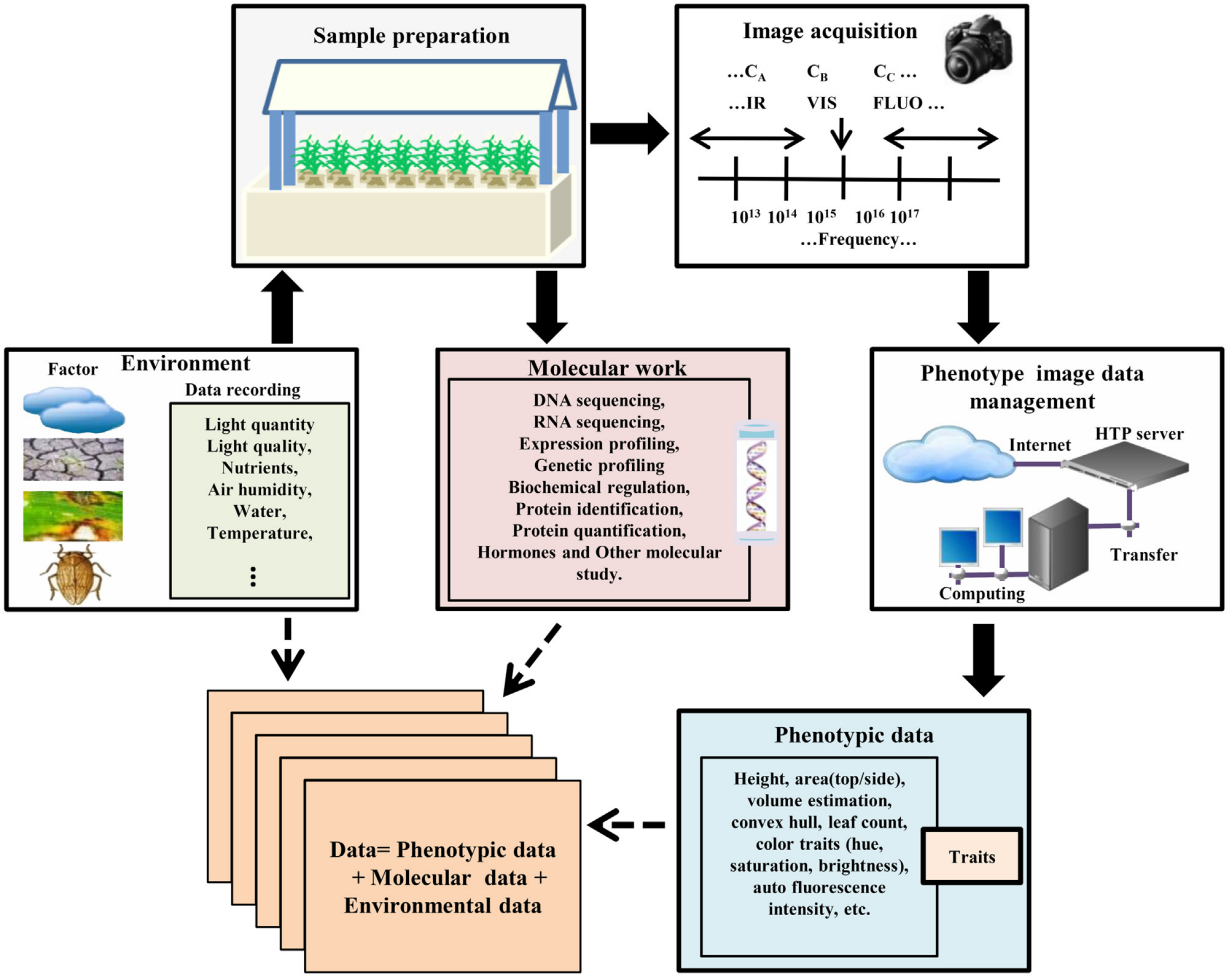
**High-throughput plant phenotyping and data accumulation.** In the phenotyping platforms for high-throughput phenotype imaging, plants are cultured under controlled environmental conditions in robotic control house systems for sample preparation. Each plant with a special treatment such as stress and/or mutant treatment is located in a container with controlled nutrient supply which is transported by the conveyor belt to the required position. The platform automatically screens germplasm resources and populations, and captures multiple top view/side view images. After image acquisition, the data should be transferred and managed by the data management system with recording environmental data and genotype information. Then image processing methods are used to calculate phenotypic traits/features from the image data. Data mining methods are used to acquire the values of the extracted features, or to statistically model and simulate the phenotype data in order to produce phenotype-genotype models in different environmental scenarios.

High-throughput experimental samples are prepared in a control phenotyping station by selecting different genotypes under normal and various treatments and conditions (**Figure 1**). The commencement and intensity of those conditions (biotic or abiotic) can be defined and controlled during the experiment. Since acquiring data must be analyzed with respect to the micro-climate and environmental conditions, it is very difficult to monitor and combine experimental materials in a dynamic process (Sadok et al., 2007; Parent et al., 2010). Advances in automation of plant phenotype, robotic- and sensor-based monitoring have enabled phenotype data acquisition, performed at regular time intervals throughout the life cycle of the plant in an automated manner for a given experiment. High-throughput phenotyping facilities of these type of experiments are commercially available, but many laboratories are now developing their own systems (Granier et al., 2006; Walter et al., 2007; Jansen et al., 2009; Skirycz et al., 2011; Tisne et al., 2013; Yang et al., 2014). Currently, various research institutes, e.g., IPK Gatersleben, Germany^2^; Crop Design, Gent, Belgium^3^; The Plant Accelerator, Adelaide, Australia^4^; PhenoArch, Montpellier, France^5^ are using these facilities. Another more advanced and dominant phenotyping platform developed by LemnaTec^6^ provides many software and tools for plant phenotype screening and image analysis.

In the modern phenotyping platforms, a fully automated control house enables plants to be delivered via conveyor belts to watering, weighing, and imaging stations, and several 100 individual plants can be imaged per day automatically. Such imaging platforms are designed by either moving plants to a stationary camera or robotically moving the camera to a stationary plant. After designing suitable experiments, high-throughput phenotyping platforms non-destructively capture multi-categories [infrared (IR), fluorescence (FLUO), visible (VIS) spectra, etc.] plant images for dissecting the phenotypic components (Brien et al., 2013; Dhondt et al., 2013; Klukas et al., 2014). In the case of *Arabidopsis*, the top view of the rosette image is sufficient for measuring rosette area (Walter et al., 2007; Skirycz et al., 2011; Tisne et al., 2013). But monocot plant morphology is complicated and the top view image alone is insufficient for morphological operation. Thus, a side view image is also required (Sozzani et al., 2014). Automated phenotyping process acquires large numbers of side-view and top-view images from different angles in regular time intervals and stores them in an image data management server (**Figure 1**).

On the other hand, image processing is one of the major tasks for acquiring accurate traits or features from these images (Hartmann et al., 2011; De Vylder et al., 2012; Klukas et al., 2014). Many general image processing software and tools are available for phenotype image processing and morphological operation of plants (Lobet et al., 2013). We illustrated a summary of phenotyping platforms and open source plant image processing and analysis software and tools in **Tables 2** and **3**, respectively.

**Table 2:** Image based automated or semi-automated high-throughput plant phenotyping platforms.

Name	URL	Description
PHENOPSIS	http://bioweb.supagro.inra.fr/phenopsis	Represents specific setups for automated phenotyping, allowing a culture of approximately 200–500 *Arabidopsis* plants in individual pots with automatic watering and imaging system (Granier et al., 2006).
WIWAM	http://wiwam.be	Like PHENOPSIS, WIWAM is an automated imaging platform simultaneously handling a large number of plants and measuring a variety of plant growth parameters with automatic watering and imaging system at regular time intervals (Skirycz et al., 2011).
PHENOSCOPE	http://www.observatoirevegetal.inra.fr/observatoirevegetal_eng/Scientific-platforms/Phenoscope	This automated phenotyping platform is an integrated device, allowing simultaneous culture of 735 individual *Arabidopsis* plants and high-throughput acquisition, storage and analysis of quality phenotypes (Tisne et al., 2013).
GROWSCREEN	http://www.fz-juelich.de/ibg/ibg-2/EN/methods_jppc/GROWSCREEN	This platform was developed to study plant leaf growth fluorescence and root architecture from seedling under control condition for visual phenotyping of large plant populations (Walter et al., 2007; Jansen et al., 2009).
TraitMill	http://www.cropdesign.com	High-throughput gene engineering platform developed by Crop Design. This is a highly versatile tool that enables large-scale transgenesis and automated high resolution phenotypic plant evolution (Reuzeau, 2007).
PHENODYN	http://bioweb.supagro.inra.fr/phenodyn	This platform monitors plant growth and transpiration rate with stressful environmental condition.
Plant Scan	http://www.csiro.au/Outcomes/FoodandAgriculture/HRPPC/PlatScan.aspx	This is an automated high-resolution phenomic center which provides non-invasive analysis of plant structure, morphology and function by utilizing cutting edge information technology including high resolution cameras and 3D reconstruction software.
LemnaTec	http://www.lemnatec.com	Visualize and analysis 2D/3D non-destructive high-throughput imaging, monitor plant growth and behavior under entirely controlled conditions in a robotic greenhouse system.
*QubitPhenomics*	http://qubitphenomics.com	Integrated conveyor and robotic high-throughput plant imaging system for the laboratory, growth chamber and field phenotype screening and phenotyping.
HRPF	N/A	High-throughput rice phenotyping facility (HRPF) designed with two main sections: rice automatic phenotyping (RAP) and yield trait scorer (YTS). This high-throughput platform was developed for automatic screening of rice germplasm resources and populations throughout the growth period and after harvest (Yang et al., 2014).

**Table 3:** Open source high-throughput plant phenotype image processing software or tools.

Name	URL	Description
ImageJ	http://imagej.nih.gov/ij	A popular, powerful, and extensible application used to process and measure a large quantity of phenotypic traits captured by images.
IAP	http://iap.ipk-gatersleben.de	Large-scale plant phenotyping image analysis software for different species based on real-time imaging data obtained from various spectra (Klukas et al., 2014).
HTPheno	http://htpheno.ipk-gatersleben.de	A high-throughput (top and side view) plant phenotyping image analysis pipeline implemented as a plug-in for ImageJ (Hartmann et al., 2011).
Rosette tracker	http://telin.ugent.be/∼jdvylder/RosetteTracker	Time-lapse visual, chlorophyll fluorescence, or thermal sequence of image analysis tool for quantification genotype effects of *Arabidopsis thaliana*, implemented as a plug-in for ImageJ (De Vylder et al., 2012).
PANorama	http://ricediversity.org	Flexible software which simultaneously measures multiple architectural and branching phenotypes from images (Crowell et al., 2014).
HPGA	http://www.msu.edu/∼jincn/HPGA	A high-throughput phenotyping tool for plant growth modeling and functional analysis (Tessmer et al., 2013).
Phenophyte	https://vphenodbs.rnet.missouri.edu/PhenoPhyte/index.php	A web-based application which measures area-related phenotypic traits from imagery and multiple experimental setup (Green et al., 2012).
SmartGrain	http://www.nias.affrc.go.jp/qtl/Smart Grain	Image analysis software for high-throughput phenotyping measurements of seed shape (Tanabata et al., 2012).
HYPOTrac	http://phytomorph.wisc.edu/HYPOTrace/download/index.htm	Automated hypocotyl growth and shape measuring software from grayscale images of *Arabidopsis* seedlings (Wang et al., 2009).
LAMINA	http://lamina.sourceforge.net	Automated leaves image analysis tool which measures a variety of characteristics related to leaf shape and size (Bylesjo et al., 2008).
Leaf Analyzer	http://leafanalyser.openillusionist.org.uk/doku.php	An automated software for rapid and large-scale analyses of leaf shape variation (Weight et al., 2008).
Leaf Processor	http://gips.group.shef.ac.uk/resources.html	An application that semi-automatically stores a number of single-metric parameters and PCA analysis for leaf shape and size including contour bending energy (Backhaus et al., 2010).

## Principles of Phenotype Data for Forecasting Plant Performance

High-throughput phenotyping provides multi-categorical phenotypic traits, and corresponding trait analysis is essential for the understanding of (a) stress resistance, (b) insect and disease resistance and for the (c) yield and quality improvement (Yang et al., 2013). The most often investigated phenotypic traits include leaf area index, biomass, canopy temperature, leaf number, seed yield, water content, leaf expansion rate, leaf shape, rate of photosynthesis, number of layers, tissue thickness, mesophyll conductance, cell size, cell division rate, and cell turgor (Tackenberg, 2007; Duan et al., 2011; Golzarian et al., 2011; Dhondt et al., 2013). The phenotype data attained by the imaging system can afford high-throughput phenotypic traits based on image color, shape, and texture (Aerts et al., 2014; Klukas et al., 2014). Color-related trait categories depend on visible/RGB cameras used for multiple phenotype images and expressed with the color intensity/pixels, and other traits depend on different geometrical and mathematical measurements, e.g., area, compactness, circumference, roundness, plant height, plant width, plant length (Klukas et al., 2014). These traits help to determine the similarity/dissimilarity among the different genotypes and treatments, different stress status and its effects on the phenotype (Chen et al., 2014b). Phenotypic features also depend on its corresponding camera being used in the phenotype imaging system, for example, fluorescence-related features, tomography-related (CT) features (Konishi et al., 2009; Aerts et al., 2014; Klukas et al., 2014; Yang et al., 2014).

Advanced mathematical and statistical methods are required to predict plant development performance using these multiple traits. For a better interpretation of results, the integration of experimental metadata within data schemas for the ensured phenotype, genomic data, and environmental data are also required. A variety of methods and tools are widely used for phenotype data analysis. A choice of statistical univariate and multivariate methods are used for hypothesis testing and measuring interrelationships among the traits. Path analysis is used to control for covariations between variables and test hypothetical causal graphs for an interpretative approach (Granier and Vile, 2014). Computer-vision based measurements and assorted data mining techniques are a more useful infrastructure for phenotype data analysis. The uses of such analytical approaches select robust genotype and describe variation of plant phenotypic characteristics, which have implications for crop development and food security (Camargo et al., 2014).

**FIGURE 2:**
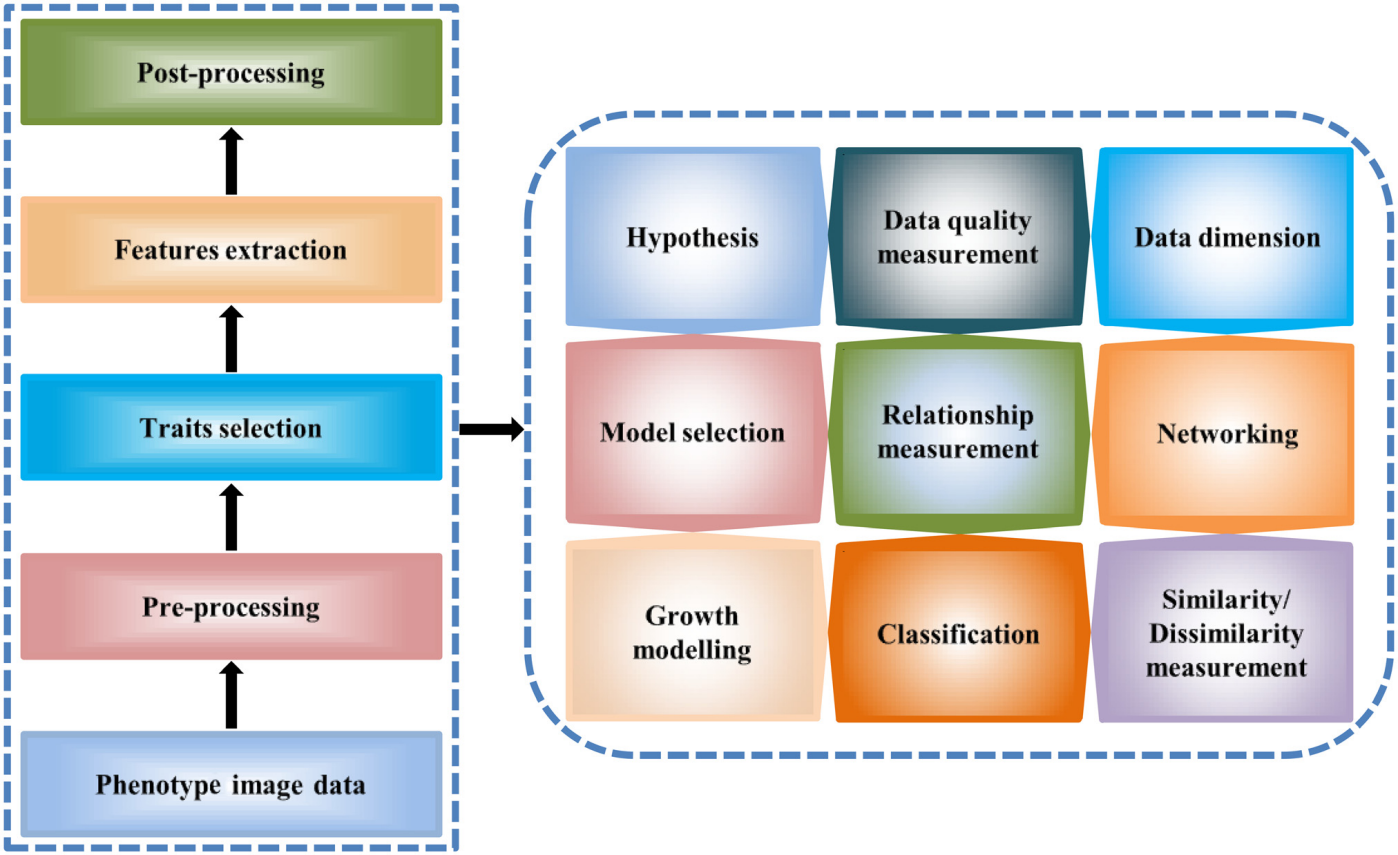
**A general workflow for the high-throughput image data analysis.** The workflow describes image data processing steps for the extraction of the quantitative traits **(left)** and the analytical methodology **(right)**.

Phenotype data analysis and modeling offer a meaningful structure of plant studies. The analysis results of phenotype data explain different relationships of traits-traits, traits-environment, phenotypic variations as well as important features for plant response, and phenotype–genotype associations. Here, we described high-throughput phenotype data analysis principles and methods (**Figure 2**) which can be of help to plant researchers to analyze large-scale phenotype image data for studying plant growth and development.

### High-Throughput Phenotype Data Analysis

Image data is pre-processed for determining quantitative or qualitative values of phenotypic traits of a plant/genotype in a given environment. Again, post-processing of phenotypic traits is also required for prediction of plant behavior using statistical analysis. Different statistical methods and algorithms are used for analyzing the process phenotype image data set, so as to move from the raw data to final results.

#### Hypothesis

Before starting the image data analysis, a proper hypothesis is required that corresponds to the expectation of the experiment within an appropriate statistical framework (Vasseur et al., 2012; Vile et al., 2012; Aerts et al., 2014).

#### Data Quality

The selection of an inadequate part of a trait often affects the data quality in a negative manner. Noisy images highly affect the dataset and could bias the results. Data normality tests and outlier detection is necessary to improve the data quality. Among the many data normalization and outlier test methods, Shapiro normality test with appropriate log-transformed and Bonferroni outlier tests are commonly used (Camargo et al., 2014). Grubb’s test is another outlier detection method that performs better for any single outlier test existing in a particular sample (Grubbs, 1950). These methods provide a powerful statistic for the data normality test and control outliers in the data set. Also, phenotype data quality is affected by throughput and image resolution (Dhondt et al., 2013).

#### Data Dimension

Phenotypic traits which are extracted from the high-throughput image dataset can be high-dimensional and be highly correlated. Therefore, analyzing this high-dimensional dataset can be difficult due to the limitations of current analytical techniques. To overcome these difficulties the data size can be reduced with as little information loss as possible. Here, we mention two statistical methods that are popular for dimensionality reduction and projection of the high-dimensional data set.

(a) PCA model (principle component analysis model): PCA identifies new features in a dataset, the principle components, which are linear combinations of the original features. Suppose that *X_nxp_* is an adjusted phenotype data matrix. Thus the basic equation of PCA is- in matrix notation- given by

$$Y=W^{T}X$$

where *Y* is a matrix of new features, called PCA, constructed as a weighted average of the original traits/features and *W* is a matrix of coefficients determined by PCA.

(b) FA model (factor analysis model): FA is another data reduction tool which removes redundancy or duplication from a set of correlated phenotypic traits. Under some assumption basic model of FA expressed as:

X=λF+e

where *X* represents observed features, *F* represents latent feature, *e* is the measurement error and is the loading value for *X*.

These statistical methods can easily select important traits and reduce the data size to explore the relationships between traits, their variations, relationships with the environmental factors, and also shows a feature’s contribution in a specific study (Vile et al., 2012).

Data analysis by these methods can be described as phenotypic variation in different conditions and enable to distinguish plants of different agronomic groups (Chen et al., 2014b). One can compute the intra-class correlation coefficients for the reliable analysis and inference to evaluate the stability of the selected features obtained from these methods (Aerts et al., 2014).

#### Model Selection

An appropriate model is needed for phenotypic variance and biomass prediction. Linear mixed-effect models can be used for phenotypic variance decomposition. Phenotypic variance decomposition results show the effect of genetic and environmental sources and their interaction for the phenotypic traits. By the likelihood estimation of mixed-effect models, it is possible to test the effect’s significance with respect to phenotypic variance (Joosen et al., 2013; Chen et al., 2014b). However, linear mixed-effect modeling approaches are more appropriate alternatives when dealing with time series data, in case observation variances are unequal or there is a degree of correlation between measurements (Camargo et al., 2014). Linear models and/or generalized linear models are widely used for biomass prediction (Golzarian et al., 2011; Camargo et al., 2014). To select the effective predictors for biomass prediction Akaike’s information criteria provides all relevant regression models (Yang et al., 2014). During the selection, it is necessary to check the heterogeneity and to solve the auto-correlation problem for phenotype data. Since, phenotype datasets may contain redundant and reproducible features and therefore, stepwise variable selection methods can be used to select an optimal set of explanatory variables for an appropriate statistical model by removing the multi-colinearity (the correlation among the independent variables of a regression model) problem among the features. Such a model provides more accurate biomass information.

#### Relationship Measurement

A bivariate relationship study is a powerful tool that describes numerous relationships among the traits-traits and traits-environment for a given genotype. This study provides the relationships between phenotypic traits, and its treatments, or other biotic and abiotic effects on the phenotype (Vasseur et al., 2012). The bivariate relationship study includes correlation of traits, allometric relationships, and QTLs relationship to demonstrate strong genetic and phenotypic relations of the same categorical traits (Chen et al., 2014b; Granier and Vile, 2014). These are also useful for measuring the genetic overlap and phenotypic similarity of different traits. A phylogenetic method within the data analysis can be used to infer the causation relationship history of both a gene and its corresponding phenotype (Kaplan and Pigliucci, 2001; Fiorani and Schurr, 2013).

#### Networking

Network analysis is also essential to find the relationships among the significant traits. To describe the network relations among the phenotypic traits, structural equation models, and Bayesian networks are used for the causal relationship and correlative network analyses, respectively. The objective of structural equation modeling is to quantify the relative contributions of correlated causal sources of variance once a certain network of interconnected features with biological significance has been accepted (Hershberger, 2001; Tisne et al., 2008). The Bayesian networks can be used to visualize genetic and/or phenotypic structure using the trait–trait genetic correlation and/or trait–trait phenotypic correlation (Chen et al., 2014b).

#### Growth Modeling

Another major part of phenotype data analysis is plant modeling (Kaitaniemi et al., 1999; Fournier and Andrieu, 2000; Buck-Sorlin, 2002; Evers et al., 2007; Buck-Sorlin et al., 2008; Xu et al., 2011). Visual 3D plant modeling and simulation provide a deeper understanding of plant growth and its relationship with the environment. Plant growth modeling helps us to test hypotheses and carry out virtual experiments concerning plant growth processes (Fourcaud et al., 2008). Functional–structural (FS) plant growth models are extremely important for integrating biological processes with environmental conditions in 3D virtual plants (Vos et al., 2010). Nowadays for more advanced research in plant sciences, time-lapse imaging-based phenotype data provides an opportunity to fit models and predict plant growth under numerous conditions. To observe the dynamic behavior of plant growth, many models have been established for different patterns of growth (Paine et al., 2012). It is well known that among the available models a sigmoid model (logistic, Gompertz) performs better for interpreting individual plant growth (Damgaard and Weiner, 2008; Karadavut et al., 2010; Chen et al., 2014b). Other population growth models, such as linear, exponential, power law, and monomolecular are also used for plant growth and pathological studies. In plant pathology, these models are often used for studying disease progression over time.

#### Classification

Classification methods are useful for biological image analysis and have simplified numerous tasks (Kamber et al., 1995; Warfield et al., 2000; Cocosco et al., 2003; Li and Chen, 2009). For example, there is a need to control diseases and numerous stresses to maintain food quality worldwide and to reduce food-borne illness originated from infected plants (Schikora et al., 2008). A wide variety of plant stress and diseases caused by the environmental factors (such as light quantity, light quality, CO_2_, nutrients, air humidity, water, temperature, drought, salinity) or other organisms (such as fungi, bacteria, and viruses) have high impact to decrease grain production and grain quality. Thus, it is important to detect and classify the plant infestations (Granier and Vile, 2014). In most cases symptoms of stress and disease in plants result is the change of the plant color. Therefore, classification approaches can be used to classify the color-related traits obtained from the plant phenotype image pixels under the biotic and abiotic conditions (Schikora et al., 2012; Chen et al., 2014b). There are many popular classification algorithms that are very helpful for plant research, such as SVM (support vector machine), Bayesian classifier, neural network (Schikora et al., 2010, 2012; Chen et al., 2014b).

#### Similarity/Dissimilarity Measurement

Clustering approaches provide important information regarding the similarity/dissimilarity among the significant features. For phenotype data analysis, these can be used to measure plant stress sensitivity between control and stress plants, phenotypic trait similarity of different genotypes, identifying unknown groups of plant species, and for supporting the idea of the phenotypic profiles corresponding to the similar genotype (Chen et al., 2014b). K-means clustering, hierarchical clustering, SOM (self-organizing map) are very popular approaches for cluster analysis of various types of dataset. Furthermore, neighbor-joining trees and phylogenetic trees are useful methods to show the phenotypic similarity and evolution of plants of various origins, revealing clusters of similar phenotypic patterns (Aerts et al., 2014). This type of analysis helps distinguish phenotypic trait’s patterns, provide important trait information and support further evaluation of the defined traits. Therefore, it is possible to find the significant association between trait profiles or pairs of the same groups or between groups of genotypes and phenotypes using correlation coefficient and test statistic (e.g., χ^2^ test, one-sided Mann–Whitney *U*-test; Aerts et al., 2014; Chen et al., 2014b).

## Conclusion and Future Indication

Research in plant biology has benefited and continues to benefit from developing high-throughput traits measurement methodologies at different levels including metabolomics, proteomics, and transcriptomics data (Granier and Vile, 2014). Advanced phenotyping technologies combine molecular techniques and non-invasive sensors with computer vision approaches. These approaches contribute to the momentous progression of high-throughput plant development research. This advanced research enables observation of high-throughput phenotypic traits and how these traits change depending on environment and genotype. These studies generate large-scale multidimensional data sets, requiring proper data management and analytical frameworks for their interpretation (Fiorani and Schurr, 2013; Klukas et al., 2014).

Most high-throughput phenotyping platforms accumulate huge amounts of image data, but these automated workflows may also increase the risk of data quality deterioration, and they might miss interesting phenotypes if proper checkpoints are not implemented at different stages of the imaging and image processing (Arvidsson et al., 2011; Dhondt et al., 2013). Therefore, it is necessary to manage and process data efficiently. Although different techniques and analytical frameworks provide a solution for handling this big data problem, these are designed individually to discuss a few specific questions and trait information (Sozzani and Benfey, 2011). Hence, the major problem is the modeling and analysis of phenotype data. There are existing statistical techniques and methods, which are often useful for dimension reduction, significant feature extraction, data pattern identification, and inference analysis (Granier et al., 2006; Karkee et al., 2009; Yang et al., 2009; Golzarian et al., 2011; Romer et al., 2011; Camargo et al., 2014).

In the near future, there is an urgent need to develop more adaptable, less expensive and sophisticated data analysis infrastructures for analyzing high-dimensional phenotype datasets in the phenomics area. In case more efficient statistical methods are being developed, multidisciplinary simulation models might support the proper experiment design and an improved acquisition of phenotype data. These aspects will support the promotion and explanation of plant growth, development, or responses to adverse environments. In this review, we have discussed different imaging techniques, phenotyping platforms, image analysis pipelines and phenotype data analysis methods for the high-throughput plant study. Based on our discussion we suggest that scientists should address the future challenges to enable the development of optimal digital phenotyping platforms. These challenges are, e.g., the reduction of phenotyping and other related laboratory costs, the development of an efficient data storage and less expensive analytical tools, as well as the improvement of the statistical methods to explore the plant dynamic phenotypic components and their properties.

## Conflict of Interest Statement

The authors declare that the research was conducted in the absence of any commercial or financial relationships that could be construed as a potential conflict of interest.

## Footnotes

1 www.fao.org

2 www.ipk-gatersleben.de

3 www.cropdesign.com

4 www.plantphenomics.org.au

5 www.phenome-fppn.fr

6 www.lemnatec.com

## References

[B1] Aerts H. J. Velazquez E. R. Leijenaar R. T. Parmar C. Grossmann P. Cavalho S. (2014). Decoding tumour phenotype by noninvasive imaging using a quantitative radiomics approach. *Nat. Commun.* 5 4006 10.1038/ncomms5006 PMC4059926 24892406

[B2] Ahmed I. M. Dai H. Zheng W. Cao F. B. Zhang G. P. Sun D. (2013). Genotypic differences in physiological characteristics in the tolerance to drought and salinity combined stress between Tibetan wild and cultivated barley. *Plant Physiol. Biochem.* 63 49–60. 10.1016/j.plaphy.2012.11.004 23232247

[B3] Arvidsson S. Perez-Rodriguez P. Mueller-Roeber B. (2011). A growth phenotyping pipeline for *Arabidopsis thaliana* integrating image analysis and rosette area modeling for robust quantification of genotype effects. *New Phytol.* 191 895–907. 10.1111/j.1469-8137.2011.03756.x 21569033

[B4] Atwell S. Huang Y. S. Vilhjalmsson B. J. Willems G. Horton M. Li Y. (2010). Genome-wide association study of 107 phenotypes in *Arabidopsis thaliana* inbred lines. *Nature* 465 627–631. 10.1038/nature08800 20336072 PMC3023908

[B5] Ayliffe M. A. Lagudah E. S. (2004). Molecular genetics of disease resistance in cereals. *Ann. Bot.* 94 765–773. 10.1093/aob/mch207 15466878 PMC4242274

[B6] Backhaus A. Kuwabara A. Bauch M. Monk N. Sanguinetti G. Fleming A. (2010). LEAFPROCESSOR: a new leaf phenotyping tool using contour bending energy and shape cluster analysis. *New Phytol.* 187 251–261. 10.1111/j.1469-8137.2010.03266.x 20456045

[B7] Baker N. R. (2008). Chlorophyll fluorescence: a probe of photosynthesis in vivo. *Ann. Rev. Plant Biol.* 64 89–113. 10.1146/annurev.arplant.59.032607.092759 18444897

[B8] Berger B. Parent B. Tester M. (2010). High-throughput shoot imaging to study drought responses. *J. Exp. Bot.* 61 3519–3528. 10.1093/jxb/erq201 20660495

[B9] Borisjuk L. Rolletschek H. Neuberger T. (2012). Surveying the plant’s world by magnetic resonance imaging. *Plant J.* 70 129–146. 10.1111/j.1365-313X.2012.04927.x 22449048

[B10] Brien C. J. Berger B. Rabie H. Tester M. (2013). Accounting for variation in designing greenhouse experiments with special reference to greenhouses containing plants on conveyor systems. *Plant Methods* 9 5 10.1186/1746-4811-9-5 PMC3630016 23391282

[B11] Brown T. B. Cheng R. Sirault X. R. Rungrat T. Murray K. D. Trtilek M. (2014). TraitCapture: genomic and environment modeling of plant phenomic data. *Curr. Opin. Plant Biol.* 18 73–79. 10.1016/j.pbi.2014.02.002 24646691

[B12] Buck-Sorlin G. (2002). “L-system model of the vegetative growth of winter barley,” in *Fifth German Workshop on Artificial Life*, eds Polani D. Kim J. Martinez T. (Lübeck: Akademische Verlagsgesellschaft Aka GmbH), 53–64.

[B13] Buck-Sorlin G. Hemmerling R. Kniemeyer O. Burema B. Kurth W. (2008). A rule-based model of barley morphogenesis, with special respect to shading and gibberellic acid signal transduction. *Ann. Bot.* 101 1109–1123. 10.1093/aob/mcm172 17766311 PMC2710269

[B14] Bylesjo M. Segura V. Soolanayakanahally R. Y. Rae A. M. Trygg J. Gustafsson P. (2008). LAMINA: a tool for rapid quantification of leaf size and shape parameters. *BMC Plant Biol.* 8:82. 10.1186/1471-2229-8-82 PMC2500018 18647399

[B15] Camargo A. Papadopoulou D. Spyropoulou Z. Vlachonasios K. Doonan J. H. Gay A. P. (2014). Objective definition of rosette shape variation using a combined computer vision and data mining approach. *PLoS ONE* 9:e96889 10.1371/journal.pone.0096889 PMC4013065 24804972

[B16] Cannon S. B. May G. D. Jackson S. A. (2009). Three sequenced legume genomes and many crop species: rich opportunities for translational genomics. *Plant Physiol.* 151 970–977. 10.1104/pp.109.144659 19759344 PMC2773077

[B17] Chen D. Chen M. Altmann T. Klukas C. (2014a). “Bridging genomics and phenomics,” in *Approaches in Integrative Bioinformatics: Towards the Virtual Cell*, Chap. 11 eds Chen M. Hofestädt R. (Berlin: Springer).

[B18] Chen D. Neumann K. Friedel S. Kilian B. Chen M. Altmann T. (2014b). Dissecting the phenotypic components of crop plant growth and drought responses based on high-throughput image analysis. *Plant Cell* 26 4636–4655. 10.1105/tpc.114.129601 25501589 PMC4311194

[B19] Chinnusamy V. Zhu J. Zhu J. K. (2007). Cold stress regulation of gene expression in plants. *Trends Plant Sci.* 12 444–451. 10.1016/j.tplants.2007.07.002 17855156

[B20] Clark R. T. Maccurdy R. B. Jung J. K. Shaff J. E. Mccouch S. R. Aneshansley D. J. (2011). Three-dimensional root phenotyping with a novel imaging and software platform. *Plant Physiol.* 156 455–465. 10.1104/pp.110.169102 21454799 PMC3177249

[B21] Cocosco C. A. Zijdenbos A. P. Evans A. C. (2003). A fully automatic and robust brain MRI tissue classification method. *Med. Image Anal.* 7 513–527. 10.1016/S1361-8415(03)00037-9 14561555

[B22] Cooper M. Van Eeuwijk F. A. Hammer G. L. Podlich D. W. Messina C. (2009). Modeling QTL for complex traits: detection and context for plant breeding. *Curr. Opin. Plant Biol.* 12 231–240. 10.1016/j.pbi.2009.01.006 19282235

[B23] Crowell S. Falcao A. X. Shah A. Wilson Z. Greenberg A. J. Mccouch S. R. (2014). High-resolution inflorescence phenotyping using a novel image-analysis pipeline, PANorama. *Plant Physiol.* 165 479–495. 10.1104/pp.114.238626 24696519 PMC4044845

[B24] Daley P. F. Raschke K. Ball J. T. Berry J. A. (1989). Topography of photosynthetic activity of leaves obtained from video images of chlorophyll fluorescence. *Plant Physiol.* 90 1233–1238. 10.1104/pp.90.4.1233 16666912 PMC1061872

[B25] Damgaard C. Weiner J. (2008). Modeling the growth of individuals in crowded plant populations. *J. Plant Ecol.* 1 111–116. 10.1093/jpe/rtn008 21237078

[B26] Dawson T. P. Jackson S. T. House J. I. Prentice I. C. Mace G. M. (2011). Beyond predictions: biodiversity conservation in a changing climate. *Science* 332 53–58. 10.1126/science.1200303 21454781

[B27] Deshmukh R. Sonah H. Patil G. Chen W. Prince S. Mutava R. (2014). Integrating omic approaches for abiotic stress tolerance in soybean. *Front. Plant Sci.* 5:244 10.3389/fpls.2014.00244 PMC4042060 24917870

[B28] De Smet I. White P. J. Bengough A. G. Dupuy L. Parizot B. Casimiro I. (2012). Analyzing lateral root development: how to move forward. *Plant Cell* 24 15–20. 10.1105/tpc.111.094292 22227890 PMC3289553

[B29] De Vylder J. Vandenbussche F. Hu Y. Philips W. Van Der Straeten D. (2012). Rosette tracker: an open source image analysis tool for automatic quantification of genotype effects. *Plant Physiol.* 160 1149–1159. 10.1104/pp.112.202762 22942389 PMC3490612

[B30] Dhondt S. Wuyts N. Inze D. (2013). Cell to whole-plant phenotyping: the best is yet to come. *Trends Plant Sci.* 18 428–439. 10.1016/j.tplants.2013.04.008 23706697

[B31] Duan L. Yang W. Huang C. Liu Q. (2011). A novel machine-vision-based facility for the automatic evaluation of yield-related traits in rice. *Plant Methods* 7 44 10.1186/1746-4811-7-44 PMC3264518 22152096

[B32] El-Lithy M. E. Clerkx E. J. Ruys G. J. Koornneef M. Vreugdenhil D. (2004). Quantitative trait locus analysis of growth-related traits in a new *Arabidopsis* recombinant inbred population. *Plant Physiol.* 135 444–458. 10.1104/pp.103.036822 15122039 PMC429397

[B33] Evers J. B. Vos J. Fournier C. Andrieu B. Chelle M. Struik P. C. (2007). An architectural model of spring wheat: evaluation of the effects of population density and shading on model parameterization and performance. *Ecol. Model.* 200 308–320. 10.1016/j.ecolmodel.2006.07.042

[B34] Finkel E. (2009). With ‘Phenomics’. Plant scientists hope to shift breeding into overdrive. *Science* 325 380–381. 19628831 10.1126/science.325_380

[B35] Fiorani F. Schurr U. (2013). Future Scenarios for Plant Phenotyping. *Ann. Rev. Plant Biol.* 64 267–291. 10.1146/annurev-arplant-050312-120137 23451789

[B36] Fourcaud T. Zhang X. Stokes A. Lambers H. Korner C. (2008). Plant growth modelling and applications: the increasing importance of plant architecture in growth models. *Ann. Bot.* 101 1053–1063. 10.1093/aob/mcn050 18387970 PMC2710283

[B37] Fournier C. Andrieu B. (2000). Dynamics of the elongation of internodes in maize (*Zea mays* L.). Effects of shade treatment on elongation patterns. *Ann. Bot.* 86 1127–1134.

[B38] Furbank R. T. (2009). Plant phenomics: from gene to form and function. *Funct. Plant Biol.* 36 5–6. 10.1071/FPv36n11_FO 32688694

[B39] Furbank R. T. Tester M. (2011). Phenomics–technologies to relieve the phenotyping bottleneck. *Trends Plant Sci.* 16 635–644. 10.1016/j.tplants.2011.09.005 22074787

[B40] Furbank R. T. Von Caemmerer S. Sheehy J. Edwards G. (2009). C-4 rice: a challenge for plant phenomics. *Funct. Plant Biol.* 36 845–856. 10.1071/FP09185 32688695

[B41] Golzarian M. R. Frick R. A. Rajendran K. Berger B. Roy S. Tester M. (2011). Accurate inference of shoot biomass from high-throughput images of cereal plants. *Plant Methods* 7 2 10.1186/1746-4811-7-2 PMC3042986 21284859

[B42] Granier C. Aguirrezabal L. Chenu K. Cookson S. J. Dauzat M. Hamard P. (2006). PHENOPSIS, an automated platform for reproducible phenotyping of plant responses to soil water deficit in *Arabidopsis thaliana* permitted the identification of an accession with low sensitivity to soil water deficit. *New Phytol.* 169 623–635. 10.1111/j.1469-8137.2005.01609.x 16411964

[B43] Granier C. Vile D. (2014). Phenotyping and beyond: modeling the relationships between traits. *Curr. Opin. Plant Biol.* 18 96–102. 10.1016/j.pbi.2014.02.009 24637194

[B44] Gratani L. (2014). Plant phenotypic plasticity in response to environmental factors. *Adv. Bot.* 2014 17.

[B45] Green J. M. Appel H. Rehrig E. M. Harnsomburana J. Chang J. F. Balint-Kurti P. (2012). PhenoPhyte: a flexible affordable method to quantify 2D phenotypes from imagery. *Plant Methods* 8 45 10.1186/1746-4811-8-45 PMC3546069 23131141

[B46] Grubbs F. E. (1950). Sample criteria for testing outlying observations. *Ann. Math. Stat.* 21 27–58. 10.1214/aoms/1177729885

[B47] Hairmansis A. Berger B. Tester M. Roy S. J. (2014). Image-based phenotyping for non-destructive screening of different salinity tolerance traits in rice. *Rice* 7 16 10.1186/s12284-014-0016-3 PMC4884049 26055997

[B48] Hartmann A. Czauderna T. Hoffmann R. Stein N. Schreiber F. (2011). HTPheno: an image analysis pipeline for high-throughput plant phenotyping. *BMC Bioinformatics* 12:148 10.1186/1471-2105-12-148 PMC3113939 21569390

[B49] Hedhly A. (2011). Sensitivity of flowering plant gametophytes to temperature fluctuations. *Environ. Exp. Bot.* 74 9–16. 10.1016/j.envexpbot.2011.03.016

[B50] Hershberger S. L. (2001). Cause and correlation in biology: a user’s guide to path analysis. Structural equations, and causal inference. *Struct. Equ. Model.* 8 646–649. 10.1207/S15328007SEM0804_08

[B51] Holtorf H. Guitton M. C. Reski R. (2002). Plant functional genomics. *Naturwissenschaften* 89 235–249. 10.1007/s00114-002-0321-3 12146788

[B52] Honsdorf N. March T. J. Berger B. Tester M. Pillen K. (2014). High-throughput phenotyping to detect drought tolerance QTL in wild barley introgression lines. *PLoS ONE* 9:e97047 10.1371/journal.pone.0097047 PMC4019662 24823485

[B53] Huang J. R. Liao H. J. Zhu Y. B. Sun J. Y. Sun Q. H. Liu X. D. (2012). Hyperspectral detection of rice damaged by rice leaf folder (*Cnaphalocrocis medinalis*). *Comput. Electron. Agric.* 82 100–107. 10.1016/j.compag.2012.01.002

[B54] Huang R. Jiang L. Zheng J. Wang T. Wang H. Huang Y. (2013). Genetic bases of rice grain shape: so many genes, so little known. *Trends Plant Sci.* 18 218–226. 10.1016/j.tplants.2012.11.001 23218902

[B55] Huang X. Wei X. Sang T. Zhao Q. Feng Q. Zhao Y. (2010). Genome-wide association studies of 14 agronomic traits in rice landraces. *Nat. Genet.* 42 961–967. 10.1038/ng.695 20972439

[B56] Jahnke S. Menzel M. I. Van Dusschoten D. Roeb G. W. Buhler J. Minwuyelet S. (2009). Combined MRI-PET dissects dynamic changes in plant structures and functions. *Plant J.* 59 634–644. 10.1111/j.1365-313X.2009.03888.x 19392708

[B57] Jansen M. Gilmer F. Biskup B. Nagel K. A. Rascher U. Fischbach A. (2009). Simultaneous phenotyping of leaf growth and chlorophyll fluorescence via GROWSCREEN FLUORO allows detection of stress tolerance in *Arabidopsis thaliana* and other rosette plants. *Funct. Plant Biol.* 36 902–914. 10.1071/FP09095 32688701

[B58] Jones A. M. Danielson J. Å. Manojkumar S. N. Lanquar V. Grossmann G. Frommer W. B. (2014). Abscisic acid dynamics in roots detected with genetically encoded FRET sensors. *Elife* 3:e01741 10.7554/eLife.01741 PMC3985517 24737862

[B59] Jones H. G. Serraj R. Loveys B. R. Xiong L. Z. Wheaton A. Price A. H. (2009). Thermal infrared imaging of crop canopies for the remote diagnosis and quantification of plant responses to water stress in the field. *Funct. Plant Biol.* 36 978–989. 10.1071/FP09123 32688709

[B60] Joosen R. V. Arends D. Li Y. Willems L. A. Keurentjes J. J. Ligterink W. (2013). Identifying genotype-by-environment interactions in the metabolism of germinating *Arabidopsis* seeds using generalized genetical genomics. *Plant Physiol.* 162 553–566. 10.1104/pp.113.216176 23606598 PMC3668052

[B61] Kaitaniemi P. Room P. M. Hanan J. S. (1999). Architecture and morphogenesis of grain sorghum, Sorghum. Bicolor (L.) Moench. *Field Crops Res.* 61 51–60. 10.1016/S0378-4290(98)00148-8

[B62] Kamber M. Shinghal R. Collins D. L. Francis G. S. Evans A. C. (1995). Model-based 3-D segmentation of multiple sclerosis lesions in magnetic resonance brain images. *IEEE Trans. Med. Imag.* 14 442–453. 10.1109/42.414608 18215848

[B63] Kaplan J. M. Pigliucci M. (2001). Genes ’for’ phenotypes: a modern history view. *Biol. Philos.* 16 189–213. 10.1023/A:1006773112047

[B64] Karadavut U. Palta C. Kokten K. Bakoglu A. (2010). Comparative study on some non-linear growth models describing leaf growth of maize. *Int. J. Agric. Biol.* 12 227–230.

[B65] Karkee M. Steward B. L. Tang L. Aziz S. A. (2009). Quantifying sub-pixel signature of paddy rice field using an artificial neural network. *Comput. Electron. Agric.* 65 65–76. 10.1016/j.compag.2008.07.009

[B66] Kastberger G. Stachl R. (2003). Infrared imaging technology and biological applications. *Behav. Res. Methods Instrum. Comput.* 35 429–439. 10.3758/BF03195520 14587551

[B67] Klukas C. Chen D. Pape J. M. (2014). Integrated analysis platform: an open-source information system for high-throughput plant phenotyping. *Plant Physiol.* 165 506–518. 10.1104/pp.113.233932 24760818 PMC4044849

[B68] Kokaly R. F. Asner G. P. Ollinger S. V. Martin M. E. Wessman C. A. (2009). Characterizing canopy biochemistry from imaging spectroscopy and its application to ecosystem studies. *Remote Sens. Environ.* 113 S78–S91. 10.1016/j.rse.2008.10.018

[B69] Konishi A. Eguchi A. Hosoi F. Omasa K. (2009). 3D monitoring spatio-temporal effects of herbicide on a whole plant using combined range and chlorophyll a fluorescence imaging. *Funct. Plant Biol.* 36 874–879. 10.1071/FP09108 32688698

[B70] Li B. Chen W. (2009). [Segmentation of multiple sclerosis lesions based on Markov random fields model for MR images]. *Sheng Wu Yi Xue Gong Cheng Xue Za Zhi* 26 861–865. 19813627

[B71] Li L. Zhang Q. Huang D. (2014). A review of imaging techniques for plant phenotyping. *Sensors (Basel)* 14 20078–20111. 10.3390/s141120078 25347588 PMC4279472

[B72] Li Y. Bock A. Haseneyer G. Korzun V. Wilde P. Schon C. C. (2011). Association analysis of frost tolerance in rye using candidate genes and phenotypic data from controlled, semi-controlled, and field phenotyping platforms. *BMC Plant Biol.* 11:146 10.1186/1471-2229-11-146 PMC3228716 22032693

[B73] Lobet G. Draye X. Perilleux C. (2013). An online database for plant image analysis software tools. *Plant Methods* 9 38 10.1186/1746-4811-9-38 PMC3853381 24107223

[B74] Lu Y. Savage L. J. Last R. L. (2011). Chloroplast phenomics: systematic phenotypic screening of chloroplast protein mutants in *Arabidopsis*. *Methods Mol. Biol.* 775 161–185. 10.1007/978-1-61779-237-3_9 21863443

[B75] Maxwell K. Johnson G. N. (2000). Chlorophyll fluorescence–a practical guide. *J. Exp. Bot.* 51 659–668. 10.1093/jexbot/51.345.659 10938857

[B76] Merlot S. Mustilli A. C. Genty B. North H. Lefebvre V. Sotta B. (2002). Use of infrared thermal imaging to isolate *Arabidopsis* mutants defective in stomatal regulation. *Plant J.* 30 601–609. 10.1046/j.1365-313X.2002.01322.x 12047634

[B77] Munns R. James R. A. Sirault X. R. Furbank R. T. Jones H. G. (2010). New phenotyping methods for screening wheat and barley for beneficial responses to water deficit. *J. Exp. Bot.* 61 3499–3507. 10.1093/jxb/erq199 20605897

[B78] Niyogi K. K. Grossman A. R. Bjorkman O. (1998). *Arabidopsis* mutants define a central role for the xanthophyll cycle in the regulation of photosynthetic energy conversion. *Plant Cell* 10 1121–1134. 10.2307/3870716 9668132 PMC144052

[B79] Okumoto S. Jones A. Frommer W. B. (2012). Quantitative imaging with fluorescent biosensors. *Ann. Rev. Plant Biol.* 64 663–706. 10.1146/annurev-arplant-042110-103745 22404462

[B80] Paine C. E. T. Marthews T. R. Vogt D. R. Purves D. Rees M. Hector A. (2012). How to fit nonlinear plant growth models and calculate growth rates: an update for ecologists. *Methods Ecol. Evol.* 3 245–256. 10.1111/j.2041-210X.2011.00155.x

[B81] Pallotta M. Schnurbusch T. Hayes J. Hay A. Baumann U. Paull J. (2014). Molecular basis of adaptation to high soil boron in wheat landraces and elite cultivars. *Nature* 51 88–91. 10.1038/nature13538 25043042

[B82] Paproki A. Sirault X. Berry S. Furbank R. Fripp J. (2012). A novel mesh processing based technique for 3D plant analysis. *BMC Plant Biol.* 12:63 10.1186/1471-2229-12-63 PMC3464618 22553969

[B83] Parent B. Suard B. Serraj R. Tardieu F. (2010). Rice leaf growth and water potential are resilient to evaporative demand and soil water deficit once the effects of root system are neutralized. *Plant Cell Environ.* 33 1256–1267. 10.1111/j.1365-3040.2010.02145.x 20302604

[B84] Pennisi E. (2008). Plant genetics: the blue revolution, drop by drop, gene by gene. *Science* 320 171–173. 10.1126/science.320.5873.171 18403686

[B85] Rajendran K. Tester M. Roy S. J. (2009). Quantifying the three main components of salinity tolerance in cereals. *Plant Cell Environ.* 32 237–249. 10.1111/j.1365-3040.2008.01916.x 19054352

[B86] Ranc N. Munos S. Xu J. Le Paslier M. C. Chauveau A. Bounon R. (2012). Genome-wide association mapping in tomato (*Solanum lycopersicum*) is possible using genome admixture of Solanum lycopersicum var. cerasiforme. *G3 (Bethesda)* 2 853–864. 10.1534/g3.112.002667 22908034 PMC3411241

[B87] Rengasamy P. (2006). World salinization with emphasis on Australia. *J. Exp. Bot.* 57 1017–1023. 10.1093/jxb/erj108 16510516

[B88] Reuzeau C. (2007). TraitMill (TM): a high throughput functional genomics platform for the phenotypic analysis of cereals. *In Vitro Cell. Dev. Biol. Anim.* 43 S4.

[B89] Romer C. Burling K. Hunsche M. Rumpf T. Noga G. Plumer L. (2011). Robust fitting of fluorescence spectra for pre-symptomatic wheat leaf rust detection with Support Vector Machines. *Comput. Electron. Agric.* 79 180–188. 10.1016/j.compag.2011.09.011

[B90] Sadok W. Naudin P. Boussuge B. Muller B. Welcker C. Tardieu F. (2007). Leaf growth rate per unit thermal time follows QTL-dependent daily patterns in hundreds of maize lines under naturally fluctuating conditions. *Plant Cell Environ.* 30 135–146. 10.1111/j.1365-3040.2006.01611.x 17238905

[B91] Salekdeh G. H. Reynolds M. Bennett J. Boyer J. (2009). Conceptual framework for drought phenotyping during molecular breeding. *Trends Plant Sci.* 14 488–496. 10.1016/j.tplants.2009.07.007 19716744

[B92] Schikora A. Carreri A. Charpentier E. Hirt H. (2008). The dark side of the salad: *Salmonella typhimurium* overcomes the innate immune response of *Arabidopsis thaliana* and shows an endopathogenic lifestyle. *PLoS ONE* 3:e2279 10.1371/journal.pone.0002279 PMC2386236 18509467

[B93] Schikora M. Neupane B. Madhogaria S. Koch W. Cremers D. Hirt H. (2012). An image classification approach to analyze the suppression of plant immunity by the human pathogen *Salmonella Typhimurium*. *BMC Bioinformatics* 13:171 10.1186/1471-2105-13-171 PMC3519609 22812426

[B94] Schikora M. Schikora A. Kogel K. Koch W. Cremers D. (2010). Probabilistic classification of disease symptoms caused by *Salmonella* on *Arabidopsis* plants. *GL Jahrestagung* 2 874–879.

[B95] Schreiber U. (1986). Detection of rapid induction kinetics with a new type of high-frequency modulated chlorophyll fluorometer. *Photosynth. Res.* 9 261–272. 10.1007/BF00029749 24442302

[B96] Skirycz A. Vandenbroucke K. Clauw P. Maleux K. De Meyer B. Dhondt S. (2011). Survival and growth of *Arabidopsis* plants given limited water are not equal. *Nat. Biotechnol.* 29 212–214. 10.1038/nbt.1800 21390020

[B97] Sozzani R. Benfey P. N. (2011). High-throughput phenotyping of multicellular organisms: finding the link between genotype and phenotype. *Genome Biol.* 12 219 10.1186/gb-2011-12-3-219 PMC3129668 21457493

[B98] Sozzani R. Busch W. Spalding E. P. Benfey P. N. (2014). Advanced imaging techniques for the study of plant growth and development. *Trends Plant Sci.* 19 304–310. 10.1016/j.tplants.2013.12.003 24434036 PMC4008707

[B99] Sticklen M. B. (2007). Feedstock crop genetic engineering for alcohol fuels. *Crop Sci.* 47 2238–2248. 10.2135/cropsci2007.04.0212

[B100] Suter L. Widmer A. (2013). Phenotypic effects of salt and heat stress over three generations in *Arabidopsis thaliana*. *PLoS ONE* 8:e80819 10.1371/journal.pone.0080819 PMC3828257 24244719

[B101] Tackenberg O. (2007). A new method for non-destructive measurement of biomass, growth rates, vertical biomass distribution and dry matter content based on digital image analysis. *Ann. Bot.* 99 777–783. 10.1093/aob/mcm009 17353204 PMC2802942

[B102] Tanabata T. Shibaya T. Hori K. Ebana K. Yano M. (2012). SmartGrain: high-throughput phenotyping software for measuring seed shape through image analysis. *Plant Physiol.* 160 1871–1880. 10.1104/pp.112.205120 23054566 PMC3510117

[B103] Tardieu F. Tuberosa R. (2010). Dissection and modeling of abiotic stress tolerance in plants. *Curr. Opin. Plant Biol.* 13 206–212. 10.1016/j.pbi.2009.12.012 20097596

[B104] Tessmer O. L. Jiao Y. Cruz J. A. Kramer D. M. Chen J. (2013). Functional approach to high-throughput plant growth analysis. *BMC Syst. Biol.* 7(Suppl. 6): S17 10.1186/1752-0509-7-S6-S17 PMC4029786 24565437

[B105] Tester M. Langridge P. (2010). Breeding technologies to increase crop production in a changing world. *Science* 327 818–822. 10.1126/science.1183700 20150489

[B106] Tisne S. Reymond M. Vile D. Fabre J. Dauzat M. Koornneef M. (2008). Combined genetic and modeling approaches reveal that epidermal cell area and number in leaves are controlled by leaf and plant developmental processes in *Arabidopsis*. *Plant Physiol.* 148 1117–1127. 10.1104/pp.108.124271 18701672 PMC2556812

[B107] Tisne S. Serrand Y. Bach L. Gilbault E. Ben Ameur R. Balasse H. (2013). Phenoscope: an automated large-scale phenotyping platform offering high spatial homogeneity. *Plant J.* 74 534–544. 10.1111/tpj.12131 23452317

[B108] Topp C. N. Iyer-Pascuzzi A. S. Anderson J. T. Lee C. R. Zurek P. R. Symonova O. (2013). 3D phenotyping and quantitative trait locus mapping identify core regions of the rice genome controlling root architecture. *Proc. Natl. Acad. Sci. U.S.A.* 110 E1695–E1704. 10.1073/pnas.1304354110 23580618 PMC3645568

[B109] Valluru R. Reynolds M. P. Salse J. (2014). Genetic and molecular bases of yield-associated traits: a translational biology approach between rice and wheat. *Theor. Appl. Genet.* 127 1463–1489. 10.1007/s00122-014-2332-9 24913362

[B110] Vasseur F. Violle C. Enquist B. J. Granier C. Vile D. (2012). A common genetic basis to the origin of the leaf economics spectrum and metabolic scaling allometry. *Ecol. Lett.* 15 1149–1157. 10.1111/j.1461-0248.2012.01839.x 22856883

[B111] Vile D. Pervent M. Belluau M. Vasseur F. Bresson J. Muller B. (2012). *Arabidopsis* growth under prolonged high temperature and water deficit: independent or interactive effects? *Plant Cell Environ.* 35 702–718. 10.1111/j.1365-3040.2011.02445.x 21988660

[B112] Vitasse Y. Bresson C. C. Kremer A. Michalet R. Delzon S. (2010). Quantifying phenological plasticity to temperature in two temperate tree species. *Funct. Ecol.* 24 1211–1218. 10.1111/j.1365-2435.2010.01748.x

[B113] Vos J. Evers J. B. Buck-Sorlin G. H. Andrieu B. Chelle M. De Visser P. H. (2010). Functional-structural plant modelling: a new versatile tool in crop science. *J. Exp. Bot.* 61 2101–2115. 10.1093/jxb/erp345 19995824

[B114] Walter A. Scharr H. Gilmer F. Zierer R. Nagel K. A. Ernst M. (2007). Dynamics of seedling growth acclimation towards altered light conditions can be quantified via GROWSCREEN: a setup and procedure designed for rapid optical phenotyping of different plant species. *New Phytol.* 174 447–455. 10.1111/j.1469-8137.2007.02002.x 17388907

[B115] Wang L. Uilecan I. V. Assadi A. H. Kozmik C. A. Spalding E. P. (2009). HYPOTrace: image analysis software for measuring hypocotyl growth and shape demonstrated on *Arabidopsis* seedlings undergoing photomorphogenesis. *Plant Physiol.* 149 1632–1637. 10.1104/pp.108.134072 19211697 PMC2663732

[B116] Wang M. Jiang N. Jia T. Leach L. Cockram J. Comadran J. (2012). Genome-wide association mapping of agronomic and morphologic traits in highly structured populations of barley cultivars. *Theor. Appl. Genet.* 124 233–246. 10.1007/s00122-011-1697-2 21915710

[B117] Warfield S. K. Kaus M. Jolesz F. A. Kikinis R. (2000). Adaptive, template moderated, spatially varying statistical classification. *Med. Image Anal.* 4 43–55. 10.1016/S1361-8415(00)00003-7 10972320

[B118] Weigel D. Mott R. (2009). The 1001 genomes project for *Arabidopsis thaliana*. *Genome Biol.* 10 107 10.1186/gb-2009-10-5-107 PMC2718507 19519932

[B119] Weight C. Parnham D. Waites R. (2008). LeafAnalyser: a computational method for rapid and large-scale analyses of leaf shape variation. *Plant J.* 53 578–586. 10.1111/j.1365-313X.2007.03330.x 18028263

[B120] Wurschum T. Maurer H. P. Kraft T. Janssen G. Nilsson C. Reif J. C. (2011). Genome-wide association mapping of agronomic traits in sugar beet. *Theor. Appl. Genet.* 123 1121–1131. 10.1007/s00122-011-1653-1 21761161

[B121] Xing Y. Zhang Q. (2010). Genetic and molecular bases of rice yield. *Ann. Rev. Plant Biol.* 64 421–442. 10.1146/annurev-arplant-042809-112209 20192739

[B122] Xu L. F. Henke M. Zhu J. Kurth W. Buck-Sorlin G. (2011). A functional-structural model of rice linking quantitative genetic information with morphological development and physiological processes. *Ann. Bot.* 107 817–828. 10.1093/aob/mcq264 21247905 PMC3077984

[B123] Yang L. N. Peng L. Zhang L. M. Zhang L. L. Yang S. S. (2009). A prediction model for population occurrence of paddy stem borer (*Scirpophaga incertulas*), based on Back Propagation Artificial Neural Network and Principal Components Analysis. *Comput. Electron. Agric.* 68 200–206. 10.1016/j.compag.2009.06.003

[B124] Yang W. Duan L. Chen G. Xiong L. Liu Q. (2013). Plant phenomics and high-throughput phenotyping: accelerating rice functional genomics using multidisciplinary technologies. *Curr. Opin. Plant Biol.* 16 180–187. 10.1016/j.pbi.2013.03.005 23578473

[B125] Yang W. Guo Z. Huang C. Duan L. Chen G. Jiang N. (2014). Combining high-throughput phenotyping and genome-wide association studies to reveal natural genetic variation in rice. *Nat. Commun.* 5 5087 10.1038/ncomms6087 PMC4214417 25295980

[B126] Yin X. Struik P. C. Kropff M. J. (2004). Role of crop physiology in predicting gene-to-phenotype relationships. *Trends Plant Sci.* 9 426–432. 10.1016/j.tplants.2004.07.007 15337492

[B127] Zhang Q. (2007). Strategies for developing Green Super Rice. *Proc. Natl. Acad. Sci. U.S.A.* 104 16402–16409. 10.1073/pnas.0708013104 17923667 PMC2034246

